# Nucleation/Growth Mechanisms and Morphological Evolution of Porous MnO_2_ Coating Deposited on Graphite for Supercapacitor

**DOI:** 10.3390/ma10101205

**Published:** 2017-10-19

**Authors:** Wenxin Huang, Jun Li, Yunhe Xu

**Affiliations:** School of Materials Engineering, Shanghai University of Engineering Science, Shanghai 201620, China; huangwenxin1993@gmail.com (W.H.); zgsdxyh@gmail.com (Y.X.)

**Keywords:** MnO_2_, coating, electrodeposition, nucleation and growth, morphological evolution

## Abstract

The nucleation and growth mechanisms of porous MnO_2_ coating deposited on graphite in MnSO_4_ solution were investigated in detail by cyclic voltammetry, chronoamperometry and scanning electron microscopy. The electrochemical properties of honeycomb-like MnO_2_ were evaluated by cycle voltammetry and galvanostatic charge-discharge. Results indicated that MnO_2_ was synthesized by the following steps: $Mn^{2+}\to Mn^{3+}+e^{-}$, $Mn^{3+}+2H_{2}O\to MnOOH+3H^{+}$, and $MnOOH\to MnO_{2}+H^{+}+e^{-}$. The deposition of MnO_2_ was divided into four stages. A short incubation period (approximately 1.5 s) was observed, prior to nucleation. The decreasing trend of the current slowed as time increased due to nucleation and MnO_2_ growth in the second stage. A huge number of nuclei were formed by instantaneous nucleation, and these nuclei grew and connected with one another at an exceedingly short time (0.5 s). In the third stage, the gaps in-between initial graphite flakes were filled with MnO_2_ until the morphology of the flakes gradually became similar to that of the MnO_2_-deposited layer. In the fourth stage, the graphite electrode was covered completely with a thick and dense layer of MnO_2_ deposits. All MnO_2_ electrodes at different deposition times obtained nearly the same specific capacitance of approximately 186 F/g, thus indicating that the specific capacitance of the electrodes is not related with deposition time.

## 1. Introduction

The supercapacitor, which is a new type of energy storage device that lies between traditional capacitors and batteries, has caused increasing concern due to its high power density, long cycle life, and wide operating temperature. Supercapacitors can be divided into two types according to storage mechanism: electric double layer capacitors and Faraday pseudocapacitors. Faraday pseudocapacitors have higher theoretical capacitance than double-layer capacitors [1]. Many electrode materials are utilized in supercapacitors, such as carbon [2,3,4], conducting polymers [5,6,7], and metal oxides [8,9,10,11]. MnO_2_ is one of the most attractive metal oxide electrode materials because of its abundance, low cost, environmental friendliness, and high theoretical capacitance of approximately 1380 F/g [12,13,14,15]. 

MnO_2_ is prepared by many techniques such as sol-gel [16,17], hydrothermal synthesis [18], precipitation [19], electrodeposition [20,21,22], and other methods [23]. MnO_2_ is synthesized in the form of powder except through electrodeposition and thus cannot be directly used as electrodes unless mixed with conductive materials (carbon black or acetylene black) and binders (polytetrafluoroethylene) in certain proportions [16,18]. A small amount of water or ethanol may be added to attain a homogenous mixture. Then, the rubber-like mixture is rolled into a film or a thin sheet or directly pressed onto a substrate, such as nickel foam, titanium mesh and stainless steel grid. Reddy et al. [16] synthesized MnO_2_ powder by sol-gel, in which the powder was mixed with carbon black (23 wt.%) and PTFE (polytetrafluoroethylene) binder (9 wt.%) before being mounted on Ti mesh to obtain the electrode. Their cyclic voltammetry (CV) test indicated that the maximum specific capacitance of the electrode was 130 F/g. Xu et al. [18] prepared α-MnO_2_ hollow spheres and hollow urchins via a simple hydrothermal process. The electrodes were fabricated by pressing mixtures containing MnO_2_ powder, acetylene black (15 wt.%), PTFE (5 wt.%), and distilled water onto nickel foams. The charge−discharge measurement showed that the largest specific capacitance of these electrodes was 167 F/g. Toupin et al. [19] synthesized α-MnO_2_ by coprecipitation. The paste was formed by mixing as-synthesized MnO_2_ powder (80 wt.%), acetylene black (7.5 wt.%), graphite (7.5 wt.%), PTFE (5 wt.%), and ethanol and pressing this mixture onto stainless steel grids to obtain the MnO_2_ electrodes. The average specific capacitance value of these electrodes was 166 F/g, as determined by CV. The abovementioned routes for MnO_2_ electrode preparation are comparatively complicated, comprising at least three steps, namely, MnO_2_ synthesis, paste preparation, and electrode assembly. Moreover, additives decrease electrode conductivity [20], and the repeatability of electrodes under these methods is difficult to control. These issues can be solved by electrodeposition. MnO_2_ electrodes can be obtained by directly depositing MnO_2_ onto substrates without conductive binder. Moreover, the applied current or potential or other parameters can be adjusted to precisely control the mass and morphology of the deposit layer, thereby enhancing the electrochemical properties of the electrode. Thus, electrodeposition has been widely used for MnO_2_ electrode acquisition. Chou et al. [24] obtained MnO_2_ electrodes by electrodepositing MnO_2_ nanowires onto a carbon nanotube paper, and a charge–discharge test showed that their specific capacitance was as high as 167.5 F/g. Babakhani et al. [25] prepared porous MnO_2_ with 1–1.5 μm diameter rods on pieces of Au-coated Si in dilute solution (0.01 M MnSO_4_ and 0.01 M Mn (CH_3_COO)_2_) via anodic deposition. The largest specific capacitance value of these MnO_2_ electrodes was nearly 185 F/g by CV. Aghazad et al. [26] synthesized MnO_2_ on a steel substrate in Mn(NO_3_)_2_ 6H_2_O via cathodic electrodeposition; the capacitive behavior was excellent such that specific capacitance was as high as 235.5 F/g. Present investigations on MnO_2_ electrodes prepared by electrodeposition focus on the influence of deposition parameters (e.g., electrolyte composition, pH value, deposition time and overpotential) on electrochemical performance. Only a few studies have investigated the nucleation and growth mechanisms of MnO_2_ deposits [1,27]. However, a clear understanding of these mechanisms offers theoretical guidance that can optimize deposition parameters, thereby improving electrochemical performance. 

In this study, porous MnO_2_ coatings were prepared on a graphite flake with MnSO_4_ electrolytes by potentiostatic deposition. The effect of deposition time on specific capacitance was investigated in Na_2_SO_4_ aqueous solution by CV and galvanostatic charge/discharge. The electrochemical oxidation process, nucleation/growth mechanism and morphological evolution of MnO_2_ were determined by CV, chronoamperometry and scanning electron microscopy (SEM).

## 2. Experimental

In this work, MnO_2_ was deposited directly onto a graphite flake (Beijing Electrical Carbon Factory, Beijing, China). These substrates (25 mm height, 10 mm width and 1 mm thickness) were degreased with acetone solution in an ultrasonic bath, and then placed in 0.1 M HCl solution at room temperature for 10 min. The graphite substrates were rinsed several times with deionized water and dried at room temperature. Finally, the substrates were weighted using a Sartorius BSA124S electronic balance (0.1 mg, Sartorius, Beijing, China).

The oxidation of Mn^2+^ in 0.14 M MnSO_4_ solution was investigated by CV on an electrochemical workstation system (CHI 660E) with a conventional three−electrode system at room temperature. A pretreated graphite flake and another that is larger than the pretreated one (25 mm height, 20 mm width and 1 mm thickness) were used as the working and the counter electrodes, respectively. The two electrodes (10 mm height) were immersed into the solution, between which a 15 mm distance was controlled. A saturated calomel electrode (SCE) was used as the reference electrode. The potential was swept from −0.2 V to 1.8 V and decreased to −0.2 V at a rate of 10 mV/s. Chronoamperometry was applied on the basis of the CV results to investigate the nucleation/growth mechanisms of MnO_2_ at certain appropriate potentials (1.3 V to 2.0 V) for 60 s.

To clearly observe the morphological evolution of MnO_2_ at different times, it was deposited at different times (0 s to 10 min) at a potential of 2.0 V. Then, the morphologies of MnO_2_ were studied by a Hitachi SU8010 SEM (Hitachi Limited, Tokyo, Japan). The phase constituents of the products deposited at 2 s and 10 min were identified using a PANalytical X’ Pert Pro X-ray diffractometer (XRD, PANalytical, Eindhoven, The Netherlands) with Cu Kα radiation (λ = 0.1540560 nm). X-ray photoelectron spectroscopy (XPS, Shimadzu/Kratos, Kyoto, Japan) was used to identify the chemical states of the elements in the products deposited at different times (2, 5, 8, 9, 20, 30, 40, and 60 s) at a potential of 2 V. XPS spectra were acquired with a Kratos Axis Ultra spectrometer using a monochromatic Al Kα source. The energy step sizes of 1 and 0.1 eV were selected for the survey and narrow-scan spectra, respectively. The binding energy scale was calibrated according to the C1s peak (at 284.8 eV) for the adventitious carbon on the analyzed sample surface.

The MnO_2_ electrodes that required electrochemical testing were weighted again after electrodeposition. The weight of graphite was subtracted to obtain the weight of MnO_2_. The electrochemical properties of the MnO_2_ deposited at different times (1, 2, 5, and 10 min) and those of the graphite without MnO_2_ were evaluated by CV and galvanostatic charge–discharge on an electrochemical workstation (CHI 600E, CH Instruments Ins, Shanghai, China). A 0.5 M Na_2_SO_4_ aqueous solution was selected as the supporting electrolyte. The scanning potential of CV ranged from −0.2 V to 0.8 V at a scan rate of 5 mV/s. Galvanostatic charge–discharge was performed in the potential range of −0.2 V to 0.8 V at a current density of 1 A/g.

## 3. Results and Discussion

### 3.1. Synthesis of MnO_2_

**Figure 1:**
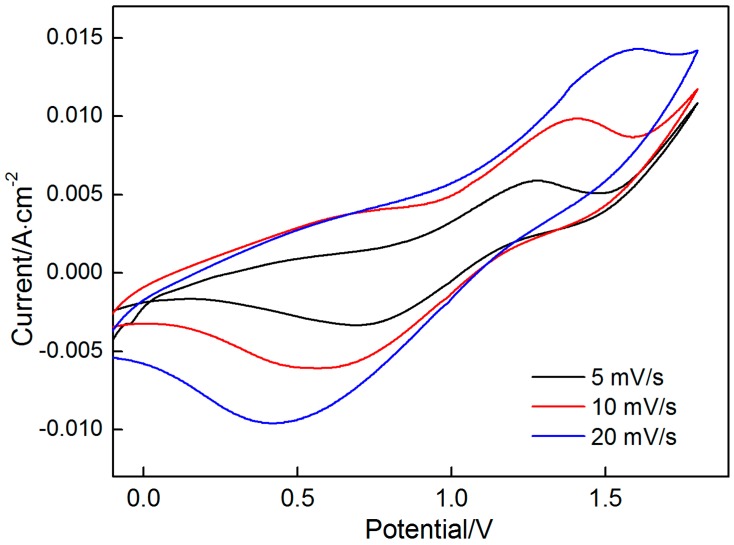
Cyclic voltammograms on graphite flake in 0.14 M MnSO_4_ electrolyte at varying scan rates of 5, 10, 20 mV/s.

**Figure 2:**
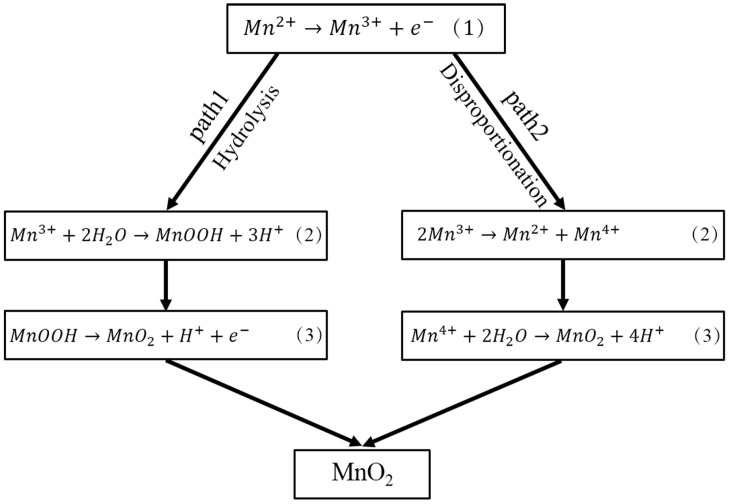
Two synthesis paths of MnO_2_ in the MnSO_4_ electrolyte.

Figure 1 shows typical CV curves with two pairs of redox peaks on a graphite flake working electrode in 0.14 M MnSO_4_ solution at varying scan rates of 5, 10, and 20 mV/s. When the scan rate is raised from 5 mV/s to 20 mV/s, the oxidation peaks move to a high oxidation potential and the reduction peaks are at the low potentials because oxidation and reduction processes deviate from the equilibrium state with increasing scan rate. During oxidation scanning, two oxidation peaks are observed. The first peak, which is at 0.5 V to 0.6 V is not noticeable; however, the second peak, which has a peak potential of approximately 1.2 V to 1.6 V is remarkably clear, thus indicating that the electrodeposition of MnO_2_ undergoes two oxidation processes. First, Mn^2+^ is oxidized to Mn^3+^ as described in reaction (1), which has been confirmed by previous studies [1,27,28]. Subsequently, Mn^3+^ is transformed into MnO_2_ by two paths (Figure 2), the choice of which is closely related to the acidic concentration in the electrolyte. Metastable Mn^3+^ ions are changed into MnOOH by reaction (2) in path 1 and precipitated on the electrode surface. Consequently, a porous layer composed of the intermediate MnOOH forms on the electrode surface. As an electronically resistive layer, MnOOH retards the diffusion of Mn^2+^ toward the electrode surface, which determines the deposition rate of MnO_2_. Then, MnOOH is oxidized to MnO_2_ by reaction (3) in path 1. Being in a highly concentrated acidic electrolyte, Mn^3+^ is stable and has sufficient time to exist and react with another Mn^3+^ to form Mn^2+^ and Mn^4+^ by reaction (2) in path 2. Then, MnO_2_ can then be rapidly formed on the electrode surface by the hydrolysis reaction of Mn^4+^ (reaction (3) in path 2). The two formation mechanisms of MnO_2_ appear different; however, they may perform mutually to result in the formation of MnO_2_. With the increase in acidic concentration, MnO_2_ prefers to be formed by path 2. The MnSO_4_ solution used in this experiment is low−concentration acidic electrolyte. Therefore, hydrolysis (path 1) dominates the formation of MnO_2_ with an accompanying disproportionation reaction (path 2).

During reduction scanning, the first reduction peak is less pronounced than the second and appears at approximately 1.45 V to 1.5 V; the second peak, which is sharper than the first, is at 0.4 V to 0.7 V. These two reduction peaks are attributed to the reduction of the deposited MnO_2_ to intermediate product MnOOH and that of MnOOH to soluble Mn^2+^ ions, which can be described by Equations (1) and (2).

(1) $$MnO_{2}+H^{+}+e^{-}\to MnOOH,$$

(2) $$MnOOH+3H^{+}+e^{-}\to Mn^{2+}+2H_{2}O,$$

### 3.2. Nucleation and Growth Mechanism

**Figure 3:**
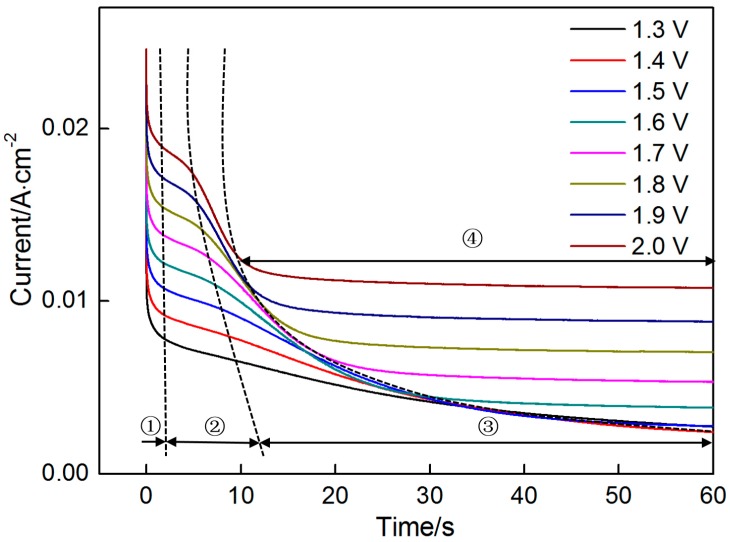
Chronoamperograms recorded on graphite flake in 0.14 M MnSO_4_ electrolyte at different step potentials ranging from 1.3 V to 2.0 V.

Chronoamperometry was conducted to explore the nucleation and growth mechanisms of MnO_2_ at different step potentials during the initial electrodeposition stage (60 s). As shown in Figure 3, a transient current is generated when the potential of 1.3 V is applied. Subsequently, the reaction is controlled by the diffusion of active ions because the electroactive species around the electrode are consumed rapidly over time. Consequently, the current gradually decrease over time. This declining trend of the current divides the process into four stages. The current drops sharply in the first stage (t < 1.5 s). In this stage, a large number of ions migrates toward the electrode and participates in the reaction occurring on the electrode surface, thereby seriously diluting active species. The current subject of the diffusion control drops quickly. Then the current significantly deviates from the original downward trend and decreases slowly overtime in the second stage (1.5 s < t < 13 s). Even a slight bump on this curve can be observed in this stage, especially at a high potential. The change in the second stage demonstrates the characteristics of the nucleation and growth of a new phase. As shown in Figure 1, MnO_2_ can be deposited at a potential of approximately 1.3 V. The increases in surface area of the electrode that arise from the nucleation and growth of MnO_2_ cause the increase in current. However, this resulting increase in current cannot compensate for the reduction in current caused by the diffusion control. Therefore, the current still decreases with time. Meanwhile, the surface area of the electrode, which increases slowly and tends to be stable, prolongs the time. The diffusion control dominates over the electrode reaction, thus causing the rapid decrease in current (t > 13 s) in the third stage. When the potential exceeds 1.5 V, a fourth stage can be observed, in which the current maintains an invariable value. It is closely related to the low conductivity of MnO_2_, as analyzed in the succeeding section.

Under planar, semi−infinite diffusion limited conditions, the relationship between current and time can be described by a Cottrell equation [29] as follows:

(3) $$i_{t}=\frac{nFAD^{1/2}C}{{\left(\pi t\right)}^{1/2}},$$

where i_t_ is the current under the diffusion-controlled process (A), *n* is the number of electrons transferred in the redox reaction, *F* is the Faraday constant, *A* is the surface area of the electrode (m^2^), *D* is the diffusion coefficient (m^2^/s), and *C* is the Mn^2+^ concentration in the bulk electrolyte (mol/m^3^). 

**Figure 4:**
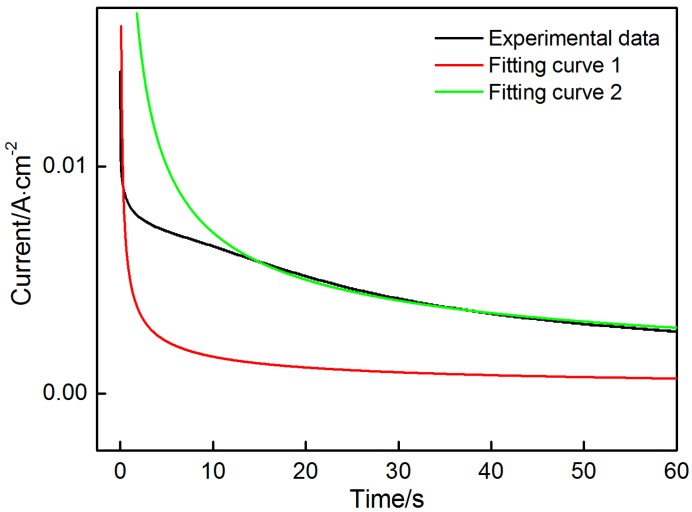
Fitting results in the first and third stages at the potential of 1.3 V.

**Figure 5:**
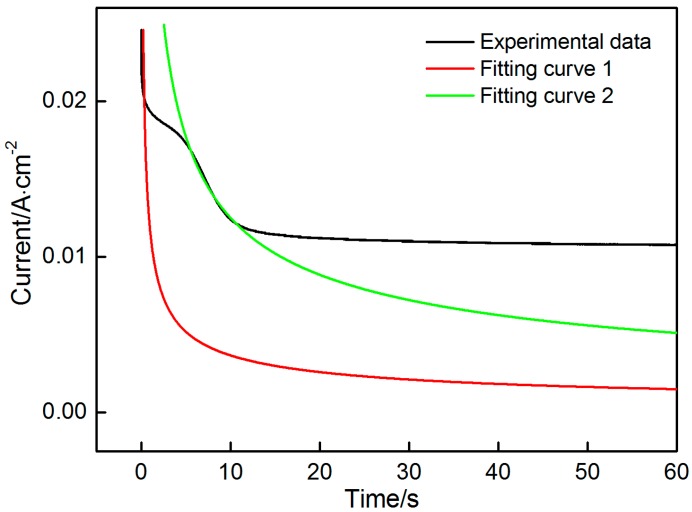
Fitting results in the first and third stages at the potential of 2.0 V.

In this system, *n*, *F*, *D,* and *C* are intrinsic parameters that are fixed in the response of current with time. However, the surface area (*A*) may change due to the nucleation, growth and connection of nuclei. In the first stage, the effect of the oxidation of Mn^2+^ to MnO_2_ on the surface area of the electrode is assumed to be negligible due to the short reaction time (<1.5 s). *A* can also be considered a constant. On the basis of these assumptions, the data obtained in the first stage are fitted into a curve, which can be precisely expressed by a Cottrell equation. Similarly, the data obtained in the third stage are also expressed by a Cottrell equation. The data obtained at the potentials of 1.3 V and 2.0 V are selected to describe the fitting process. As shown in Figure 4 and Figure 5, the fitting results are as follows:

The potential is 1.3 V:
The first stage (Fitting curve 1): $i_{t\;}=\;{0.01298t}^{-1/2}$, $\frac{nFAD^{1/2}C}{\pi }=0.01298$
The third stage (Fitting curve 2): $i_{t}=0.05658t^{-1/2}$, $\frac{nFAD^{1/2}C}{\pi }=0.05658$
The potential is 2.0 V:
The first stage (Fitting curve 1): $i_{t}\;=\;{0.02928t}^{-1/2}$, $\frac{nFAD^{1/2}C}{\pi }=0.02928$
The third stage (Fitting curve 2): ${\;i}_{t}=0.1911t^{-1/2}$, $\frac{nFAD^{1/2}C}{\pi }=0.1911$

The experiment data are in good agreement with a Cottrell equation. Except *A*, the parameters remain constant throughout the deposition in the first and third stages. Therefore the ratio of the two coefficients in the two equations can reflect the change in active surface area. As shown in Table 1, the surface area of the electrode shows a threefold increase after the deposition of MnO_2_, thus indicating that a porous MnO_2_ layer is formed on the surface of the electrode. The porous layer with large surface area contributes to the enhancement of the charge storage. 

With the increase in step voltage, some noticeable changes can be observed. (i) The curves in the second stage demonstrate an increasingly upward trend, and the duration is shortened; (ii) The decline in current is intensified with time in the third stage, and the duration is also shortened; (iii) When the applied potential is higher than 1.5 V, the fourth stage, in which the current maintains a constant value, can be clearly observed. 

The deposition rate strongly depends on the potential. An increase in potential enhances the nucleation and growth rate. Therefore, the characteristics of nucleation and growth in the second stage become increasingly apparent with increasing potential. The increased nucleation and growth rate mean that a big surface area can be obtained in a short time. The increased deposition rate accelerates the reduction in surface area that results from the connection of nuclei. Moreover, the reaction is controlled by the diffusion of active species around the electrode. The combined effects of the above mentioned factors cause a violent decline in current with increasing potential. Over time, a layer of MnO_2_ with a comparatively stable surface area forms on the electrode surface, thus shielding the entire electrode surface and reducing the drive force for the migration and oxidation of Mn^2+^ with its poor electronic conductivity. That is, the reaction is not controlled by the diffusion of ions. Consequently, the current tends to be stable. The duration in the third stage is closely related to the final surface area of MnO_2_. The denser the formed MnO_2_, the shorter the duration of the third stage. As shown in Table 1, the active area ratios before and after the deposition of MnO_2_ decreases with increasing potential, thereby indicating that denser MnO_2_ forms at higher potentials. This finding explains the shortened duration with increasing potential in the third stage.

Instantaneous nucleation and progressive nucleation are the two significant nucleation processes. In the instantaneous process, nuclei are formed at nearly the same time and the growth of nuclei is dominant in the subsequent reactions. In progressive nucleation, nuclei are constantly formed not only on the substrate surface but also on previously formed nuclei during the entire deposition, accompanied with the sustained growth of nuclei. Instantaneous nucleation achieves better crystalline quality than progressive nucleation [30]. Hills deduced two equations about the relationship between current and time for judging the nucleation process on the surface of the electrode.

For instantaneous nucleation:

(4) $$i_{\left(t\right)}=\frac{zFN_{o}\pi {\left(2DC\right)}^{3/2}M^{1/2}}{\rho^{1/2}}t^{1/2},$$

for progressive nucleation:

(5) $$i_{\left(t\right)}=\frac{2zFK_{n}N_{o}\pi {\left(2DC\right)}^{3/2}M^{1/2}}{3\rho^{1/2}}t^{3/2},$$

where *z* is the valence number, *N*_0_ is the initial nucleation number, *D* is the diffusion coefficient for the deposited ions (cm^2^/s), *C* is the bulk concentration of the deposited ions (mol/cm^3^), *M* is the atomic weight of the deposits (g/mol), *ρ* is the density of the deposits (g/cm^3^), *K_n_* is the nucleation constant, i_(t)_ is the corresponding current when time is t (A), and *t* is the polarization time (s).

**Figure 6:**
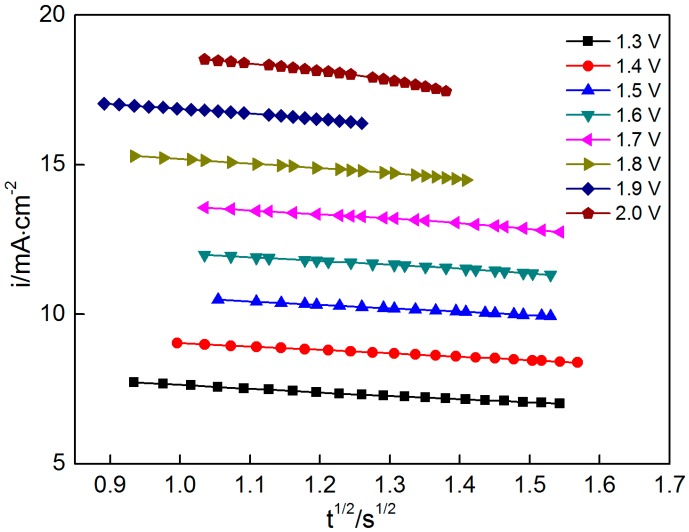
Current i as a function of t^1/2^ at various potentials ranging from 1.3 V to 2.0 V.

**Figure 7:**
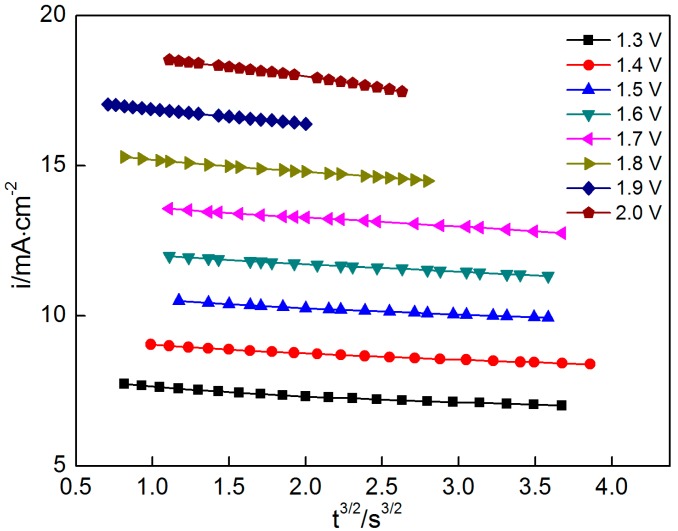
Current i as a function of t^3/2^ at various potentials ranging from 1.3 V to 2.0 V.

The above equations show that the current exhibits a linear relationship with t^1/2^ and t^3/2^ under instantaneous nucleation and progressive nucleation, respectively (Figure 6 and Figure 7, respectively). The experiment data in the second stage (Figure 3) are selected for exploring the nucleation mechanism. The results indicate that good linearity can be obtained in i versus t^1/2^ and t^3/2^ at all potentials. However, the theoretical curves cannot be plotted because many parameters, such as *N*_0_ and *C*, in Equations (4) and (5) cannot be obtained. Consequently, the nucleation mechanism cannot be confirmed precisely by comparing the theoretical and experimental curves.

Scharifker–Hills (SH) model was used to investigate the nuclear mechanism as parameters can be crossed out effectively by normalizing i and t to the peak current i_m_ and peak time t_m_, respectively.

For instantaneous nucleation:

(6) $${\left(i/i_{m}\right)}^{2}=\frac{1.9542}{t/t_{m}}{\left\{1-\exp\left[-1.2564\left(t/t_{m}\right)\right]\right\}}^{2},$$

For progressive nucleation:

(7) $${\left(i/i_{m}\right)}^{2}=\frac{1.2254}{t/t_{m}}{\left\{1-\exp\left[-2.3367\left(t/t_{m}\right)\right]\right\}}^{2},$$

**Figure 8:**
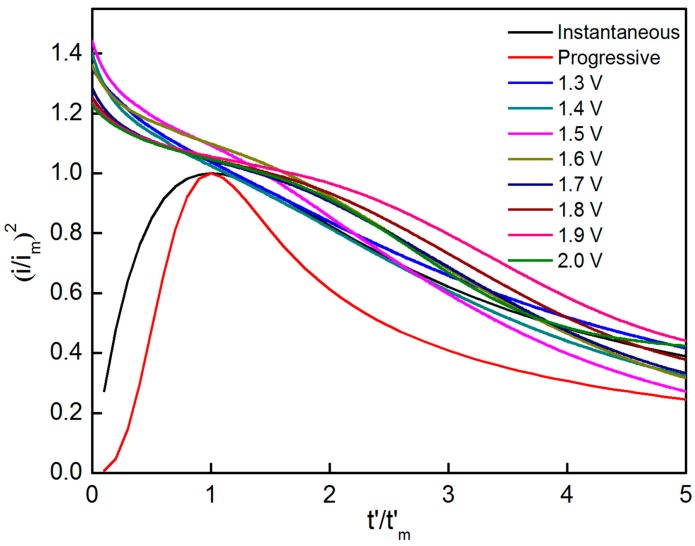
Comparison of dimensionless (i/i_m_)^2^ vs. t’/t’_m_ plots of nucleation process on graphite flake at various potentials (1.3 V to 2.0 V).

A short incubation time (t_0_) occurs prior to nucleation, thus possibly interfering with the theoretical curve. Thus, t_0_ is subtracted to obtain correct the normalization curve, and t and t_m_ are redefined by $\;t\prime =t-t_{0}$ and $t_{m}{}^{\prime }=t_{m}-t_{0}$. The two theoretical curves for instantaneous and progressive nucleation and the eight experimental curves for the different potentials are shown in Figure 8. Experimental data obtained in the second stage seriously deviate from the theoretical curves due to factors that are mainly connected to the change in current over time. As shown in Figure 3, the current slowly increases with time and no obvious current peaks are apparent at the second stage. These findings might have resulted from the low potential, low Mn^2+^ concentration and weak MnO_2_ conductivity. Data obtained in the third stage agree with the instantaneous nucleation model. Results further indicate that instantaneous nucleation dominates the MnO_2_ nucleation process_._

### 3.3. Morphological Evolution of MnO_2_

**Figure 9:**
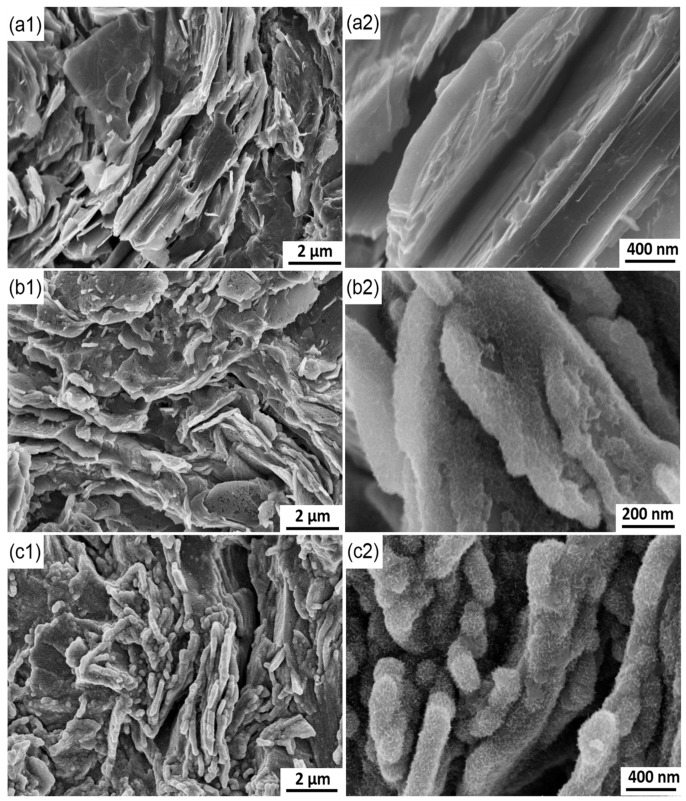
SEM images of the electrode surface at different deposition time. (**a**) 0 s; (**b**) 2 s; (**c**) 5 s.

Chronoamperometry was used to indirectly investigate MnO_2_ nucleation and growth, in particular, by analyzing current–time response at different potentials. To fully understand the deposition process, SEM was used to observe the morphological evolution of MnO_2_ deposited at 2 V at different times. On the basis of the analysis of the chronoamperometry data, SEM images are also divided into three groups corresponding to three stages. Figure 9 shows the morphologies of the deposited MnO_2_ in the initial stages (0, 2 and 5 s). Prior to MnO_2_ deposition, the initial graphite electrode comprises several flakes in different directions (Figure 9(a1)). Many gaps can be observed clearly among them, and the whole surface looks rough. White patterns are found on the surfaces of the flakes in high-resolution SEM images, thus revealing that the surfaces of the flakes are not level (Figure 9(a2)). These morphological characteristics contribute to the heterogeneous nucleation due to the high specific surface area. When the deposition time is set to 2 s, the morphology of the electrode is similar to that before the deposition (Figure 9(b1)). However, a thin and dense layer of deposits can be found on the surfaces of the flakes upon close examination. Except for a few scattered zones with a radius of approximately 50 nm, the zones are nearly covered with the deposits. CV and chronoamperometry data confirm that the deposits are MnO_2_. Figure 9(b2) further reveals that the deposits comprise fine equiaxed grains with a size of approximately 20 nm. Moreover, the grain boundaries look brighter than the grains, resulting in the formation of a honeycomb-like structure. According to imaging theory of secondary electron, the grain boundaries protrude from the surface of the deposited layer. 

Chronoamperometry results indicate that nucleation occurs at approximately 1.5 s (Figure 3). Then, nuclei grow and connect over time, thus finally resulting in the formation of a dense layer of MnO_2_. However, the two above steps are completed in a short time (approximately 0.5 s) in this experiment (Figure 9(b2)). A large number of nuclei is formed in nearly every zone of all the graphite flakes exposed to the electrolyte due to the rough surfaces of those flakes (Figure 9(a2)). These nuclei are expected to grow omnidirectionally along the three-dimensional directions parallel and perpendicular to the flake surface. The number of nuclei is too large, thereby reducing the space for lateral growth. After an exceedingly short time, the lateral growth is stopped as the grains are connected with one another, thus resulting in the formation of a dense layer of MnO_2_. Then, the vertical growth of grains becomes predominant. Compared with the grains, grain boundaries are preferred growth sites due to their high energy. Consequently, flaky MnO_2_ with honeycomb-like structure grows along the grain boundaries. However, the grain boundaries will not always grow in preference to the grains. If the grain boundaries lengthen, the electronic resistance between growth sites and substrate will be increased in the vertical direction due to the poor electronic conductivity of MnO_2_. This increased resistance causes a reduction in the overpotential on the grain boundaries. The deposition rate of MnO_2_ in the grain boundaries will be gradually reduced to even less than that of the grains over time. Depending on the interplay of the two changes in energy for MnO_2_ growth and electronic conductivity of MnO_2_, grains and grain boundaries compete with each other, thus resulting in an increasing quantity of MnO_2_ deposited on the electrode. As shown in Figure 9(c1), the morphology of the electrode after 5 s is significantly different from those (0 and 2 s). Many gaps that formed on the original graphite flakes are now filled with MnO_2_ such that the entire surface looks relatively leveled. Moreover, compared with the other zones, the edges of the graphite sheets are covered with a thicker layer of MnO_2_ due to the edge effect. The charge density on the electrode edge is higher than that in the other zones and is proportional to the electric field strength. Therefore, an increasing number of particles tends to be deposited around the electrode edge under a strong electric field. During the deposition, the growth rates in different zones vary. Some zones with high growth rates become spherical particles, which may connect with one another, thus forming strip-like particles.

**Figure 10:**
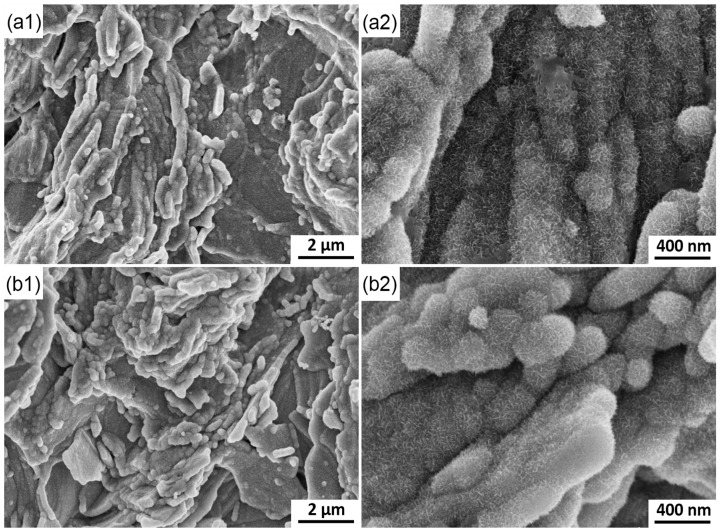
SEM images of the electrode surface at different deposition time. (**a**) 8 s; (**b**) 9 s.

Two durations (8 and 9 s) are selected for the deposition of MnO_2_ in the third stage (Figure 4). In Figure 10, the gaps among the graphite flakes are less and the surface is more leveled than those in Figure 9(c1). The initial surface is barely distinguishable, especially at 9 s, when the electrode has been thoroughly covered with a thick layer of MnO_2_. Meanwhile, a large number of spherical particles and elongated strips can still be seen (Figure 10(a2,b2)).

**Figure 11:**
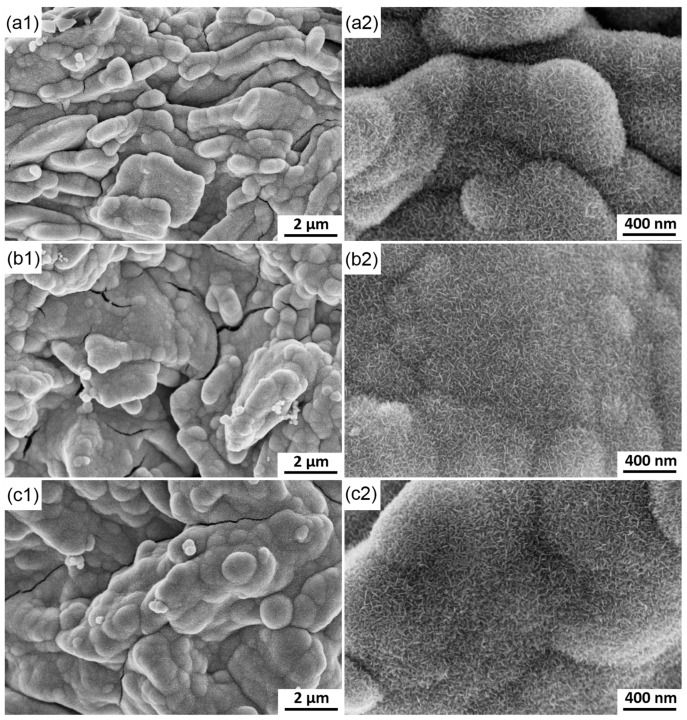
SEM images of the electrode surface at different deposition time. (**a**) 20 s; (**b**) 30 s; (**c**) 40 s.

When the deposition time is further prolonged in the fourth stage, the morphology of MnO_2_ exhibits a significant change (Figure 11). Certain spherical and strip-like particles further connect with one another to form big block-like particles. New gaps begin to form among different particles due to their different growth rates. With increased deposition time, the ions in the electrolyte become difficult to diffuse into the gaps, thus accelerating the formation of gaps. Meanwhile, fine particles may be swallowed by coarse particles. The morphology of the electrode exhibits nearly no change at 20, 30 and 40 s. 

Observation of the MnO_2_ deposited at different times can reasonably explain the change in current with time (Figure 3). No evident current peaks are observed in the second stage, and this finding is attributed to the instantaneous nucleation and initial morphological characteristic of the electrode. Numerous nuclei form on the rough surfaces of the graphite flakes and connect with one another in an exceedingly short time, thus leaving insufficient time for the current to respond. Meanwhile, MnO_2_ has poor electronic conductivity, thereby reducing the deposition rate and prevents the current from increasing. With increased deposition time, many gas among the flakes are filled with MnO_2_, thereby significantly reducing the areas. The current is decreased correspondingly at the third stage. The increase in electronic conductivity of the deposited layer further restrains the current. The surface area in the third stage does not significantly change due to the gaps in the original graphite flakes filled with MnO_2_ deposits. When the electrode is covered with a thick layer of MnO_2_ with high electronic resistance, the deposition rate is significantly decreased and the process is not controlled by the diffusion of ions. The current remains nearly constant in the fourth stage.

When the deposition potential is constant, the deposition process of MnO_2_ (including nucleation and growth) changes constantly with the deposition time due to the changes in the deposition environment around the substrate (including deposit thickness, pH, and concentration of active Mn^2+^). The thicker the deposit layer, the lower the deposition rate, which results from the poor conductivity of MnO_2_. The concentration polarization resulting from the depletion of Mn^2+^ around the graphite substrate can also lead to a decrease in deposition rate. Besides the two factors, pH is the other significant factor that affects the deposition process of MnO_2_. According to the synthetic paths of MnO_2_ (Figure 2), a large number of H^+^ is generated during the formation of MnO_2_. Therefore, a low concentration of H^+^ (higher pH) is beneficial to producing MnO_2_. High pH values will result in the formation of a large number of nuclei, thereby causing an increasing size of substrate surface zone to be covered by MnO_2_. That is, a continuous coating is expected at high pH values. However, an increasing quantity of H^+^ is generated and rich in the solution close to the substrate surface with increasing deposition time, thus inhibiting the formation and decreasing the deposition rate of MnO_2_. Moreover, pH value is closely related to the electrostatic interaction between the graphite substrate and MnO_2_, which also has a non–negligible effect on the deposition process [31]. The iso−electric point of graphite is approximately 5.0 [32], whereas that of MnO_2_ is approximately 3.6 [33], which means that MnO_2_ precipitates more easily on the surface of the graphite substrate when the pH value of the solution ranges from 3.6 to 5.0. Prior to the deposition of MnO_2_, the pH value of the solution is approximately 3.2. The pH value further decreases along the deposition of MnO_2_ due to the increase in concentration of H^+^. That is, a repulsive force nearly appears between the graphite substrate and MnO_2_ during deposition. Compared with the driving force resulting from the overpotential applied to the graphite substrate, the repulsive force is low enough for MnO_2_ to still be deposited.

### 3.4. XRD and XPS Results

**Figure 12:**
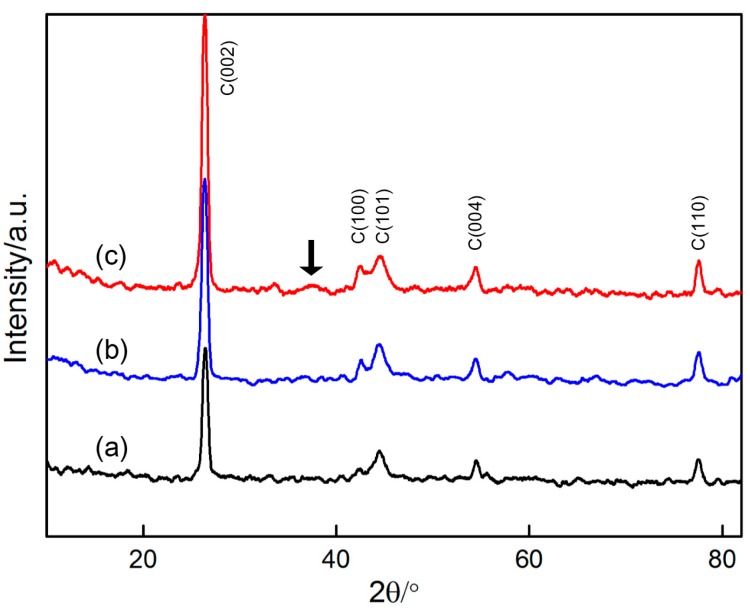
XRD patterns of: (**a**) graphite substrate; (**b**) graphite substrate covered with MnO_2_ (deposited for 2 s); (**c**) graphite substrate covered with MnO_2_ (deposited for 10 min).

Figure 12 shows the XRD patterns of the graphite substrate when bare and when covered with MnO_2_ deposited at maximum and minimum deposition times (2 s and 10 min). The patterns of the three samples are similar. Five strong diffraction peaks associated with the graphite substrate (JCPDS card no. 00-001-0640) can be clearly seen, thus indicating that the graphite from the substrate is the main phase before and after the deposition of MnO_2_. When the deposition time is short (2 s), MnO_2_ cannot be detected from the pattern. When the time is increased to 10 min, a broadened peak with low intensity can be observed at approximately 37° (marked by arrows). The weak peak can be identified from the contribution of the deposited MnO_2_ [34].

**Figure 13:**
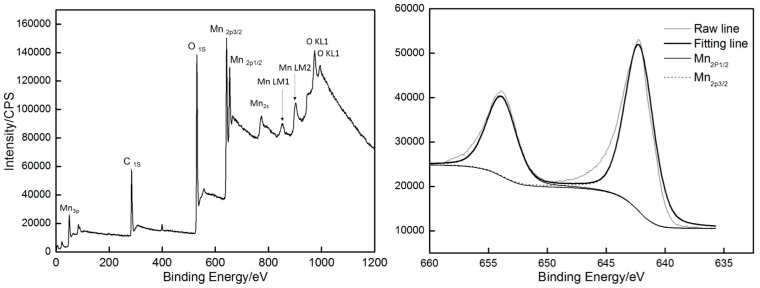
The survey and Mn_2p_ XPS spectrum of the deposit (60 s).

XPS was used to identify the deposit formed on the electrode surface. The XPS survey spectrum of the deposit (60 s) (Figure 13) shows that the deposit is mainly composed of three elements (Mn, O, C). C may be coming from the substrate, and the deposit can be confirmed as MnO_2_. Narrow spectrum was also conducted to determine the chemical valence state of Mn in the deposit. Figure 13 also shows that all Mn_2p_ spectra consist of two strong peaks, corresponding to Mn_2p3/2_ and Mn_2p1/2_ with bonding energies of 642.2 eV and 653.8 eV, respectively. They are agree with those of Mn^4+^ in MnO_2_, thus suggesting that Mn^2+^ ions in the electrolyte are oxidized into MnO_2_ during deposition.

**Figure 14:**
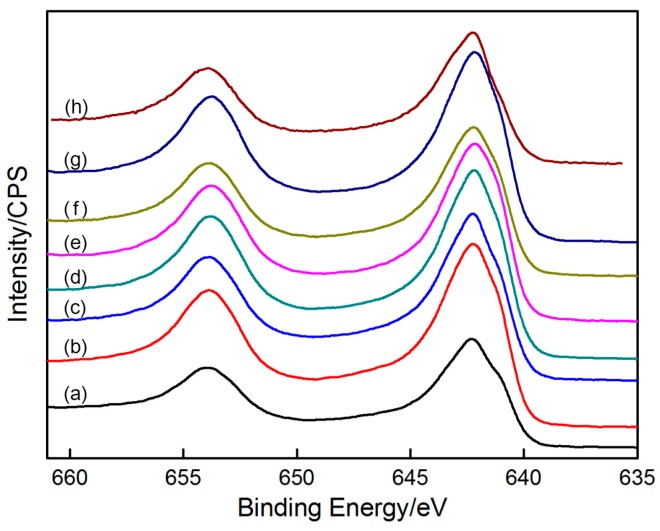
Mn_2p_ spectra of MnO_2_ deposited for different times: (**a**) 2 s; (**b**) 5 s; (**c**) 8 s; (**d**) 9 s; (**e**) 20 s; (**f**) 30 s; (**g**) 40 s; (**h**) 60 s.

In addition, the XPS spectra of the deposits at different deposition times (2, 5, 8, 9, 20, 30, 40, and 60 s) were tested (Figure 14). Two strong peaks can be clearly seen in each spectrum, and their bonding energies exhibit nearly no change with the increase in deposition time. The analysis of the bonding energies of Mn_2p3/2_ and Mn_2p1/2_, proves the existence of MnO_2_, thereby indicating that the phase constituents of the deposits do not change with increasing deposition time. It can be also found that MnO_2_ was deposited on the graphite substrate by the instantaneous nucleation model in an extremely short time (2 s).

### 3.5. Electrochemical Performance

**Figure 15:**
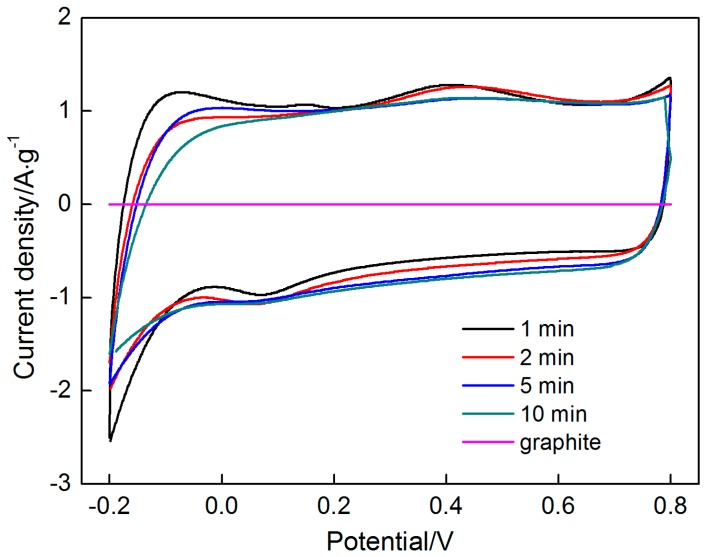
Cyclic voltammograms of graphite and the MnO_2_-based electrode at different deposition time from 1 min to 10 min.

Figure 15 shows the CV curves of the graphite and the MnO_2_ electrode at different deposition times (1, 2, 5, and 10 min) in a potential range of −0.2 V to 0.8 V. The CV curve of the graphite is nearly an overlapping line whose integral area can be negligible and shows that the capacitance of graphite is small. The other CV profiles are nearly overlapping and present a rectangular shape with good symmetry. A pair of redox peaks are hardly noticeable in each CV curve. The current is nearly constant with the increase in potential, thus suggesting that the MnO_2_ electrode is charged and discharged at a constant rate. Moreover, both ends of the curves show that the current rises or decreases nearly instantaneously when the scanning direction is changed, thereby indicating that the charging and discharging processes present good dynamic reversibility. These characteristics indicate that all MnO_2_ electrodes demonstrate an ideal capacitive behavior. As pseudocapacitive electrodes, the MnO_2_ electrodes exhibit the pseudocapacitive behavior resulting from the surface redox reaction as described in Equation (8) [26,35].

(8) $$MnO_{2}+X^{+}+e^{-}↔MnOOX^{+},$$

where *X^+^* represents *H^+^* or *Na^+^* in the supporting electrolyte. The intercalation and deintercalation of *X^+^* are accompanied by the reduction and oxidation, respectively.

The specific capacitance values can be calculated by integrating the area of the CV curves as follows:

(9) $$C=\frac{1}{mv\left(∆V\right)}\int_{V_{a}}^{V_{c}}I\left(V\right)dV,$$

where *C* is the specific capacitance (F/g), Δ*V* is the potential range, m is the mass of MnO_2_ (g), *v* is the scan rate (V/s), and *I*(*V*) is the current response. 

The specific capacitance values of 187, 184, 189 and 184 F/g were calculated using Equation (9) at the deposition times of 1, 2, 5, and 10 min, respectively, thus indicating that MnO_2_-based electrodes have good supercapacitive behavior. Notably, the capacitance values of these MnO_2_ electrodes are nearly identical. As analyzed above, the deposition rate of MnO_2_ is low due to the high electric resistance of the thick layer of MnO_2_, which has poor conductivity. The deposition mass does not significantly change at the four deposition times. Moreover, the morphologies of the MnO_2_ deposited at the four deposition times are also similar. Therefore, their specific capacitances are approximately the same.

**Figure 16:**
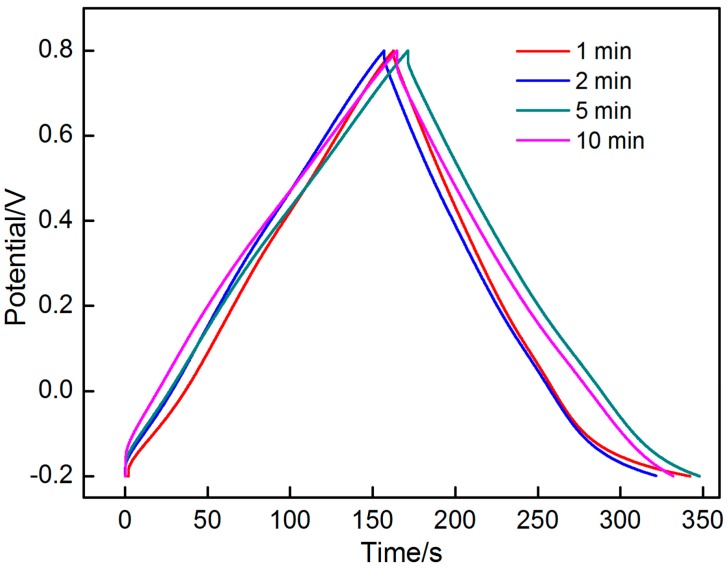
Galvanostatic charge/discharge profiles of the MnO_2_-based electrode at different deposition time from 1 min to 10 min.

The specific capacitances of these MnO_2_ electrodes were measured by galvanostatic charge–discharge. As shown in Figure 16, the four charge–discharge curves at the different deposition times exhibit a typical symmetrical triangular shape, thus indicating that the MnO_2_ electrodes possess good charge−discharge performance and the reversibility.

The specific capacitance values can be calculated as follows:

(10) $$C=\frac{I\times ∆t}{m\times ∆V^{\prime }}$$

where *I* is the applied constant current (A), *m* is the mass of MnO_2_, Δ*V* is the potential window during cycling and Δ*t* is the time of cycle (s).

The specific capacitance values of the different deposition times are 179, 167, 177 and 169 F/g, which suggest the same regularity as that of the result of CV.

## 4. Conclusions

1. MnO_2_ was deposited on flaky graphite by electrochemical oxidation in 0.14 M MnSO_4_. The reaction process could be divided into three steps: $Mn^{2+}\to Mn^{3+}+e^{-}$; $Mn^{3+}+2H_{2}O\to MnOOH+3H^{+}$; and $MnOOH\to MnO_{2}+H^{+}+e^{-}$.
2. The deposition of MnO_2_ proceeded in four stages, as shown by chronoamperometry and SEM observations. After a very short incubation time of approximately 1.5 s (the first stage), a large number of nuclei formed on nearly all graphite flakes by instantaneous nucleation, grew, and connected with one another in a remarkably short time of 0.5 s. MnO_2_ with a grain size of approximately 20 nm showed a honeycomb-like morphology. As grain boundaries and grains grew coordinately, spherical and strip-like particles formed on some zones, especially on the edges of flakes. The initial morphology of the graphite was still maintained in the second stage. Additional MnO_2_ was deposited on the graphite surface in the third stage, thus making the initial morphology of the graphite hardly distinguishable. The graphite was completely covered with a thick and dense layer of MnO_2_ with stable morphology in the fourth stage.
3. Finally, the layer of honeycomb-like MnO_2_ with large surface area (approximately four times that of the electrode before the deposition) possessed a high specific capacitance (CV: approximately 186 F/g; galvanostatic charge–discharge: approximately 173 F/g. In addition, the specific capacitance had no relationship with the deposition time.

## Figures and Tables

**Table 1:** Fitting results and active area ratios before and after MnO_2_ deposition.

Potential	1.3 V	1.4 V	1.5 V	1.6 V	1.7 V	1.8 V	1.9 V	2.0 V
$A_{1}=\frac{nFAD^{1/2}C}{\pi }$ (Fitting curve 1)	0.0130	0.0152	0.0177	0.0199	0.0222	0.0246	0.0269	0.0293
$A_{2}=\frac{nFAD^{1/2}C}{\pi }$ (Fitting curve 2)	0.0566	0.0647	0.0711	0.0780	0.0814	0.0875	0.0932	0.0998
*A*_2_/*A*_1_	4.36	4.26	4.02	3.92	3.66	3.56	3.46	3.41
